# Cultural Adaptation and Validation of the Inflammatory Bowel Disease Disability Index in a Spanish Population and Its Association with Sociodemographic and Clinical Factors

**DOI:** 10.3390/ijerph16040635

**Published:** 2019-02-21

**Authors:** Rafael López-Cortés, Raquel Herrero-Hahn, Rosanna De la Rosa-Eduardo, Rafael Montoya-Juárez, María Paz García-Caro, Blanca Marín-Fernández, César Hueso-Montoro

**Affiliations:** 1 Ministry of Health, Andalusian Goverment, 18014 Granada, Spain; rafael.lopez.cortes@juntadeandalucia.es; 2 Faculty of Health Sciences, University of Granada, 18016 Granada, Spain; raquelherrero@correo.ugr.es (R.H.-H.); mpazgc@ugr.es (M.P.G.-C.); 3 Department of Health Sciences, Public University of Navarra, 31008 Pamplona, Spain; rosanna.delarosa@unavarra.es (R.D.l.R.-E.); blanca.marin@unavarra.es (B.M.-F.); 4 Faculty of Health Sciences, University of Jaén, 23071 Jaén, Spain; chueso@ujaen.es

**Keywords:** inflammatory bowel disease, disability, quality of life

## Abstract

Inflammatory bowel diseases generate disability. We aimed to adapt and validate the Inflammatory Bowel Disease Disability Index in a Spanish population and to analyze the sociodemographic and clinical factors associated with disability in patients with Crohn’s disease and ulcerative colitis. Cultural adaptation and validation of psychometric properties in the index were done, along with an observational, cross-sectional, and analytical approach to determine associations with sociodemographic and clinical factors. Sociodemographic data, quality of life (using the Inflammatory Bowel Disease Questionnaire-32), and indicators of disease activity were collected, among others. A total of 170 subjects participated. The index showed high internal consistency, with a Cronbach’s alpha of 0.869 and concurrent validity with the Inflammatory Bowel Disease Questionnaire-32 (*r* = 0.723, *p* < 0.001). The average score of the index was −3.91. Greater degrees of disability were found in women (mean = −6.77) than in men (mean = −1.25) (*p* = 0.018), in patients with Crohn’s disease (mean = −5.94) rather than those with ulcerative colitis (mean = −0.94) (*p* = 0.028), and in patients in the moderately active disease phase (mean = −20.94) rather than those in the mildly active disease phase (mean = −2.65) and/or those in remission (mean = −1.40) (*p* < 0.001). The Disability Index is a valid tool for the Spanish population and is associated with sex, type of illness, and disease activity. It is a useful index in evaluating and monitoring disability in patients with inflammatory bowel disease.

## 1. Introduction

Currently, inflammatory bowel diseases (IBDs) are chronic pathologies that can be considered to be disabling since they can negatively affect the functional status of patients and have physical, labor, economic, and psychological repercussions [1]. In fact, people with IBD have a greater disability than the general population, in addition to a marked deterioration in health-related quality of life [2].

Disability is a common challenge of global health, and interest in it has been increasing over the past three decades. In addition, the treatment paradigm for IBD has changed considerably, from the control of symptoms to total control of the disease to prevent structural damage and disability [3].

Since the 1990s, attention has been focused mainly on quality of life with IBD [3]. However, quality of life is a subjective concept and is linked to the individual personality of each subject [4], while disability is an objective determination, understood as the human experience related to bodily functions and deteriorated structures, limitations on activity, and restrictions on participation in the interaction with environmental factors [5]. That is, disability is an objective way of measuring the impediments experienced in health areas or domains different from the concept of quality of life [6].

Therefore, it is necessary to change the way in which we approach disability in patients with IBD, since this approach has the potential to improve self-management and the satisfaction of people with these diseases [7]. Furthermore, it is essential to prevent the progression of the disease and to limit its clinical and disabling impacts [8].

In a recent systematic review performed to assess the incidence and prevalence of IBD worldwide, it was concluded that at the beginning of the 21st century, IBD became a global disease with an increasing incidence in recently industrialized countries whose societies had become westernized [9]. For this reason, IBD has become a global disease [10]. The estimates made for Spain have indicated incidences of 7.5 cases per 100,000 inhabitants for Crohn’s disease (CD) and 9.1 cases per 100,000 inhabitants for ulcerative colitis (UC) [11].

There have been few studies that have addressed the disabling effects and life experiences of people with these pathologies compared to other chronic inflammatory diseases, such as multiple sclerosis [12]. In recent years, the need for additional research to better quantify disability in these patients has been noted [13]. 

In this context, the Inflammatory Bowel Disease Disability Index (IBDDI) was created. The IBDDI was the first specific index used as an instrument to measure disability from IBD [12] and was developed based on the International Classification of Functioning Disability and Health (ICF) developed by the WHO6 together with an extensive search of the areas of disability in this type of pathology [14]. In recent years, research results have been published that support this index as a valid tool for measuring disability in both CD and UC [15,16]. Likewise, the index has demonstrated its usefulness as a predictor of the direct costs of healthcare for these diseases, confirming an increase in cost as self-reported disability worsens within the IBDDI [17].

A systematic review with a recent meta-analysis found IBDDI versions adapted from the original to French, Portuguese, Australian, Dutch, Belgian, Canadian, and Singaporean populations, concluding that the index is a reliable and valid questionnaire, but that more studies are needed to measure its response capacity and interpretability [18]. This review did not include any version adapted and validated in the Spanish population.

The main objective of this research was to perform a cross-cultural adaptation to obtain a Spanish version of the IBDDI and then to analyze its psychometric properties in the Spanish population. In addition, we analyzed what sociodemographic and clinical factors were associated with disability measured through the index.

## 2. Materials and Methods 

A study of cultural adaptation and validation of psychometric properties of the index was conducted, together with an observational, cross-sectional, and analytical study to determine the sociodemographic and clinical factors associated with disability.

### 2.1. Subjects

The study population included patients older than 18 years of age, diagnosed with CD or UC, who attended the digestive pathology outpatient clinic at the Hospital Complex of Granada, located in the southeast of Spain, and the Digestive Service of the Hospital Complex of Navarra, in the northern zone of the country. The inclusion criteria were a confirmed medical diagnosis of CD or UC, an agreement to participate in the study endorsed with a signature indicating informed consent, and the language ability to complete the data collection forms.

To determine the number of subjects needed to perform a factorial analysis, which was the procedure used for construct validity, the guidelines of Beavers et al. [19] were followed, which recommend that regardless of the number of subjects per variable, the sample must be at least 150 subjects.

Participants were recruited through consultation with specialists in digestive pathology, who were responsible for informing them of the implementation of the study and inviting the subject to participate. A systematic sampling strategy was performed, where the participation of alternate patients was requested according to the order in which they were cited and attended to. To do this, each day of data collection began with the first subject mentioned, and from there was the alternate selection. If accepted, the patient went to an additional consultation where he or she was fully informed of the study. At that time, it was verified that he or she met the inclusion criteria, and the data were collected. This procedure was repeated every day during the medical consultation to reach the sample size, so that the period of data collection was from September 2014 to April 2015.

The final sample consisted of 170 patients, 95 in the Hospital Complex of Granada and 75 in the Digestive Service of the Hospital Complex of Navarra.

### 2.2. Cultural Adaptation and Psychometric Validation

The original IBDDI [12] is composed of 19 items divided into 28 questions that fall into five ICF categories: (1) General Health, (2) Body Function, (3) Body Structures, (4) Participation Activity, and (5) Environmental Factors. The index was designed to explore the severity of disability and limitations in the following areas: Sleep; state of mind; abdominal pain; bowel movement frequency; regular defecation; participation in social events and work or school; and aggravating effects of medicines, food, family, and health professionals.

A study of cultural adaptation and validation of scales was performed, following the guidelines proposed by Beaton et al. [20]. First, the original questionnaire in English was translated into the target language (Spanish). This translation was created by two bilingual translators previously informed of the objectives of the research and without any contact between them. Two independent translations were obtained. After synthesizing the two translations, back-translation was performed.

Next, a review was conducted by a committee of experts with experience in patients with IBD and by experts in linguistics who evaluated the validity of the content, appearance, and semantic equivalence between these versions and the original.

Finally, a pilot test (pretest) was conducted with 22 subjects with IBD that served to establish whether the questionnaire could be satisfactorily understood and completed by the patients and to estimate the time required for its completion. The pilot study included patients who met the inclusion criteria described for the study population and were recruited through intentional sampling from the members of the Inflammatory Bowel Disease of Granada Association and from patients under treatment at the Granada Hospital. These subjects were not included in the final sample. It was assumed that the items that were referred to as difficult to understand by 15% or more of the participants would be modified [21], although in the pilot test, no item met this condition. For this reason, none of the items initially proposed was modified.

The final Spanish version of the index is available in the “Supplementary Table S1”.

Several analyses were conducted to evaluate the psychometric aspects of the instrument. Reliability was estimated by calculating Cronbach’s alpha for the complete index and evaluating the change caused by removing each of the items from it. To determine the validity of the construct, the Kaiser–Meyer–Olkin test and the Bartlett sphericity test were performed to verify if it was possible to submit the index to a subsequent factorial analysis. For the feasibility of the index, the time needed to complete it and an evaluation, the simplicity of the format, and the clarity of the items were assessed and evaluated through a review by the judges’ committee and the pilot test. For concurrent validity, the IBDDI was correlated with the Inflammatory Bowel Disease Questionnaire-32 (IBDQ-32) [22], which is used as the gold standard for determining the quality of life in patients with IBD, and its validated version adapted into Spanish [23].

### 2.3. Procedure for the Analysis of Associated Factors

An observational, cross-sectional, and analytical study was performed. The variables studied were sex, age, age at diagnosis, level of schooling, work status, marital status, type of disease (CD or UC), smoking status, progression of the disease (this variable was categorized according to the criteria of Bernklev et al. [24] and Jägulth et al. [25]), and index of activity of the disease, measured with the Harvey–Bradshaw Index [26] for CD and the Truelove–Witts Index adapted for UC [27,28]. This latter variable was analyzed as a categorical variable following the proposal of both indices, which are analogous in the categorization of their respective scores in four phases of the disease: Remission or inactivity, mildly active, moderately active, and severe.

The numerical scoring system used for the IBDDI was the one elaborated and implemented by Leong et al. [16]. The response to each item in the index could be scored as dichotomous (yes/no), as an ordinal on a Likert scale of 1–5, or as a numerical value (for example, body mass index). The scores of each question were combined in the totals by domain, and an overall score was obtained, resulting in degrees of disability ranging from −80 (maximum degree of disability) to 22 (without disability), with 0 representing the point of neutrality. That is, more negative scores indicated a greater disability. To determine the degree of disability, these authors established a correlation with the ability to work. Specifically, severity was defined according to the percentage of actual hours worked in the previous week, as follows: Minimum (100%), slight (76–99%), moderate (50–75%), and severe (<50%). These percentages were correlated with the numerical scores of the IBDDI, as shown below (Severity of Disability according to the IBDDI): Minimum (> −10), Medium (−10 to −19), Moderate (−20 to −35), Severe (≤ −35).

Regarding data analysis, univariate analysis was performed by means of calculation of the means and standard deviations for quantitative variables, while qualitative variables were expressed as absolute frequencies and percentages. For bivariate analysis, the Kruskal–Wallis test was used when the contrast variables were qualitative polycotomic, the Mann–Whitney *U* test was used when the contrast variables were dichotomous qualitative, and the Spearman correlation was used for quantitative contrast variables. Nonparametric techniques were used, since a normality analysis performed with the Shapiro–Wilk test on the IBDDI variable showed a non-normal distribution. The values of this variable are expressed as medians, in addition to means and standard deviations. 

Additionally, several multiple linear regression models were designed to analyze the relationships between sociodemographic and clinical characteristics in relation to the IBDDI score. Specifically, a global model was made, which was accompanied by models differentiated by sex and type of disease. These last two models were developed after the global model was not fitted. We included as factors those variables that generated statistically significant differences in the bivariate analysis or those that were conceptually considered relevant in light of the reviewed bibliography. In addition, the sample size was taken into account to obtain an approximate number of 15 observations per variable included in the model, as recommended in the multivariate analysis. The variables included were age at diagnosis, smoking habit, quality of life, activity index, sex, work status, and type of illness. For each model, the fitting conditions were checked.

In all of the analyses, *p* < 0.05 was considered significant. Statistical processing was performed with the Statistical Package for the Social Sciences (SPSS) version 22 program (SPSS Inc., Chicago, IL, USA) for Windows and R Commander (R version 3.2.2, https://www.r-project.org/, Spanish R-UCA Project, http://knuth.uca.es/R).

### 2.4. Data Collection Procedure

A data collection notebook was prepared that included the sociodemographic and clinical variables form, the IBDQ-32, the adapted version of the IBDDI, all of the documentation referring to ethical considerations, and the protocolized information on the procedure. The purpose was for researchers from Granada and Pamplona to perform the same process and to avoid biases associated with the researcher at the time of data collection. Two researchers who had the protocol and received specific training collected the data. The time for data collection was 25–30 min.

### 2.5. Ethical Considerations

The ethical principles for medical research with human beings were applied [29]. An informational sheet and an informed consent form were prepared. All participants signed informed consent forms before being included in the investigation. This research was approved by the Clinical Research Ethics Committee of Navarra and the Coordinating Committee of Ethics of Biomedical Research of Andalusia and Granada. Likewise, to preserve the anonymity of the participants, each data collection notebook was identified with an alphanumeric code.

## 3. Results

### 3.1. Characteristics of the Subjects

Of the 170 subjects who participated in the study, 51.8% were men, and 48.2% were women. The average age was 44.31 years. In total, 59.4% of the subjects were diagnosed with CD and 40.6% with UC; and 54.1% of the subjects were in the remission or inactivity phase of the disease, followed by 35.3% in the mildly active disease phase and 10.6% in the moderately active disease phase. None of the subjects were in the severe phase. Note that 78.2% of the sample claimed not to smoke. The average score of the IBDQ-32 was 167.38. The average score of the IBDDI was −3.91 (median = −1). A total of 67.6% of the sample presented a minimum intensity of disability, while 20.6% were mild, 7.1% were moderate, and 4.7% were severe (Table 1).

### 3.2. Results of the Cultural Adaptation Phase and Psychometric Validation

The index appeared to measure what was intended in both the pilot test and the expert committee. The participants also stated that the items were relevant and easy to understand.

In terms of internal consistency, a Cronbach’s alpha value of 0.869 was obtained, which ranged between 0.856 and 0.876 when eliminating each of the items in the index.

The construct validity was determined by factor analysis. The Kaiser–Meyer–Olkin (KMO) test and the Bartlett sphericity test were performed to verify if it was possible to submit the scale to factor analysis, resulting in satisfactory tests (KMO = 0.853, χ^2^ = 2424.79, *p* < 0.001). In the exploratory factor analysis, the factor extraction method used was principal component analysis, along with the Varimax rotation method. The results showed the existence of a total of six factors that explained 60.24% of the total variance (Table 2): Factor 1 included functional patterns in IBD (items 1 to 13); Factor 2 aspects of the patient’s environment that negatively influence the activity of the disease, bodily functions, and the activities of daily life (worsen items 14, 15, 16, and 17); Factor 3 aspects of the patient’s environment that positively influence the activity of the disease, bodily functions, and activities of daily life (relieve or improve items 14, 15, 16, and 17); Factor 4 structure and functions related to digestion (items b515 and s540); Factor 5 the social services and health system (items 18 and 19); and Factor 6 musculoskeletal structures related to movement and functions related to defecation and digestion (items b515, b525, and s770).

The average time to complete the questionnaire was 15 min. There were no objections from the committee of judges or from the members of the pilot test in relation to the other feasibility criteria.

**Figure 1:**
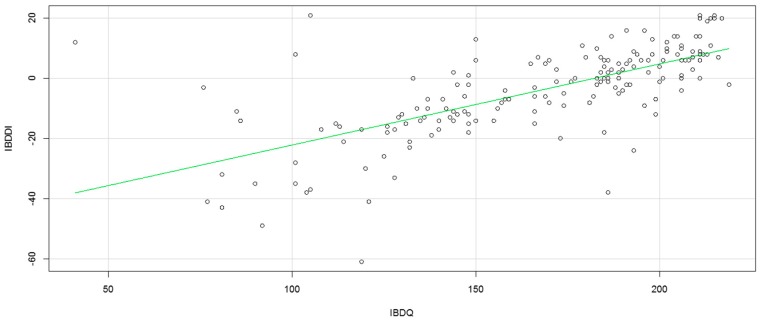
Concurrent validity, Inflammatory Bowel Disease Disability Index, and Inflammatory Bowel Disease Questionnaire-32 correlation.

Finally, to determine the concurrent validity, the IBDDI and the IBDQ-32 were correlated, showing a direct and moderate association (although with a coefficient very close to the estimated value for strong correlation) between both instruments (*r* = 0.723, *p* < 0.001; Figure 1).

### 3.3. Results of the Analysis of Associated Factors

Statistically significant differences were found in the IBDDI score according to sex, type of IBD, and activity index. Men presented an average score of −1.25 (median = 0.00) compared to women, with a score of −6.77 (median = −5.50) (*p* = 0.018). In relation to the type of disease, subjects with CD presented a mean score of −5.94 (median = −3.00), while the mean score was −0.94 (median = 2.00) in subjects with UC (*p* = 0.028). Statistically significant differences were also found when comparing the index score to the three activity index categories that were described in the sample. The differences were found between the group of subjects in the moderately active disease phase, which presented an average of −20.94 (median = −18.00), and the remaining two groups: The subjects in the remission or inactivity phase of the disease presented an average of −1.40 (median = 0.00), and the group of subjects in the mildly active phase of the disease had a mean of −2.65 (median = −0.50) (*p* < 0.001 for both) (Table 3).

In the correlation analysis, no association of the index was found with age (*r* = −0.135, *p* = 0.080). However, there was a slightly significant inverse association with the age at diagnosis (*r* = −0.160, *p* = 0.037).

Regarding the multivariate analysis, the variables included in the global model generated for the IBDDI explained 47% of the variability, although this did not meet the fit criteria. The sample was then divided by sex and type of disease, since these factors were significant in the bivariate analysis and were conceptually variables of interest in light of the literature reviewed. We obtained fitted models for men (*R*^2^ = 72%, Table 4) and UC (*R*^2^ = 76%, Table 5): The models were not fitted for women and CD. In the case of men, the multivariate analysis confirmed the association of the activity index with the IBDDI, and being a smoker appeared to be an associated factor in this population. In the case of the UC model, the association observed between the IBDDI and work status stood out. Both models confirmed a strong association between the IBDDI and the IBDQ-32.

## 4. Discussion

The Spanish version of the IBDDI had a high internal consistency with stability since the elimination of any of the items on the scale did not cause a large change in this value. In addition, the factorial structure showed a distribution of the items in six factors. Although there was a certain similarity with the structure used to design the original scale [12], differences in the number of factors and distribution of items have also been observed in relation to other adaptation and validation studies published for other populations. More studies are needed along this line to determine the factorial structure of the instrument, although it is also possible to think about the possibility of different structures depending on the population, shaped by implicit differences in each population. 

The IBDDI has been validated in a French population and showed good psychometric properties. It should be noted that during the validation process, the scale was reduced to 14 items from the initial 28, and the total score ranged from 0 to 100 points [15]. Another similar study conducted in a Portuguese population also reduced the final version of the IBDDI to 14 items and obtained a Cronbach’s alpha of 0.88 [30]. A study developed in Australia, from which the scoring system for our research was taken, showed that the IBDDI had a high internal consistency (Cronbach’s alpha of 0.94, somewhat higher than that of our study), also showing a good discriminating capacity [16]. In the Netherlands, the index has been validated and showed a Cronbach’s alpha of 0.872 [17]. Finally, it is worth highlighting a systematic review with a meta-analysis that brought together different versions of the IBDDI, confirming the good internal consistencies of the index in all of the studies analyzed [18].

Another finding to highlight was the agreement found between the IBDDI and the IBDQ-32, with a worse quality of life associated with greater disability, as demonstrated by other studies [16,31,32]. These results demonstrate the usefulness and complementarity of both instruments in the comprehensive evaluation of patients with IBD, each one in its specific construct.

Regarding the analysis of factors associated with disability measured with the IBDDI, the results showed a significant difference according to the type of IBD: People with CD had a greater disability than subjects with UC. However, the study conducted in the French population did not describe any significant difference in the scores according to the type of disease [15]. In line with the results observed in our population, we found a study conducted in the Dutch population [17] and the aforementioned systematic review, which concluded that these differences do exist in favor of patients with CD, although considerable heterogeneity was found among the studies analyzed [18].

With regard to the influence of sex, data were found that showed more disability in women than in men, with a difference of approximately six points. This finding is in line with the studies conducted in the French and Portuguese populations, which also observed worse IBDDI scores in women than in men [15,30]. It should be noted that the scoring system used was not the same in both studies. In contrast, a study conducted in the Australian population [16] found that younger age, but not sex, was associated with more disability. A Dutch study also did not observe differences according to sex [17].

Disability has been shown to be sensitive to the index of disease activity: The IBDDI scores increased as the intensity of the disease increased, with clear differences between the subjects who were in the moderately active phase of the disease compared to those who were in the mildly active, remission, or inactivity phases. In this case, in addition, the group of subjects with a moderately active disease had moderate levels of disability, while the other two groups had minimal levels of intensity. The multivariate analysis in the fitted models also corroborated this finding. The literature reviewed supports this result, evidencing an association between disability and the situation of activity or inactivity in the disease [15,16,17,30,32]. Specifically, the systematic review mentioned above showed that patients with an active disease had higher rates of disability than those in remission, although it is again worth noting the high heterogeneity found among the studies reviewed [18]. 

Finally, a significant correlation was observed, albeit slight, between the IBDDI score and the age at diagnosis, although this association was not confirmed when comparing the index to the progression of the disease, meaning the finding was inconclusive based on our results. It would be interesting to explore this association in future research, since it is likely that there is a curvilinear relationship between these variables. When comparing this to other investigations, we again found diverse results. A previous study identified a significantly higher risk of developing disabilities at five years after the initial diagnosis and among patients under 40 years of age with CD [33]. Another study stated that being younger is a predictor of the presence of disabilities [34]. However, the aforementioned systematic review showed that age or age at the time of diagnosis in most studies were not related factors [18].

This study had some limitations. More studies are needed that use the validated version of the disability index for IBD as a measuring instrument to confirm structural validity and to evaluate the response capacity of the IBDDI as a predictor of disability from disease. The sample size used in this study was sufficient to determine the main psychometric properties of the index but was insufficient to propose more complex factor analyses relevant to the processes of adaptation and validation of health measurement instruments. Furthermore, future research with more subjects will help to determine the associations found in our study between disability measure with the IBDDI and sociodemographic and clinical variables.

Furthermore, it should be noted that patients were selected from outpatient care in the review and follow-up consultation. The question is whether differences could be found in the IBDDI data between extrahospital and hospitalized patients. In this sense, and to avoid biases, the determination of the activity of the disease was introduced according to indexes of clinical activity recognized for use in medical research. Moreover, in these types of pathologies, the type of healthcare is not as different as the degree of real activity that the disease presents in each subject.

## 5. Conclusions

In light of the results obtained, it is concluded that the Spanish version of the IBDDI had good psychometric properties, with a factorial structure based on six factors and showing a moderate/high agreement with the quality of life measured with the IBDQ-32.

Having a version of the IBDDI validated in the Spanish population is an important advance for the evaluation of disability in patients with IBD, as it will be able to better determine the needs and social and functional limitations faced by affected individuals. Furthermore, the instrument can also be applicable in the clinical context, allowing health professionals to perform thorough and comprehensive patient assessments, including in their management parameters related to not only signs and symptoms but also to the disability caused. Finally, the applicability for research is evident because it opens up the possibility of conducting comparative studies between populations, as validated versions already exist in a significant number of countries.

## Figures and Tables

**Table 1:** Sociodemographic and clinical variables.

**Quantitative Variables**	**Min–Max**	**Mean ± SD**
*Age*	16–80	44.31 ± 14.20
*Age at diagnosis*	8–72	32.91 ± 13.44
*IBDDI* ^1^	−61–(+)21	−3.91 ± 15.02
*IBDQ-32* ^2^	41–219	167.38 ± 37.94
**Categorical variables**	***N* (170)**	**(%)**
*Sex*		
Male	88	51.8
Woman	82	48.2
*Level of schooling*		
Basic education, primary or none	24	14.1
Middle or secondary education	30	17.6
Complete secondary or secondary education	62	36.5
University education	54	31.8
*Employment status*		
Working	92	54.1
Unemployed	37	21.8
Retired	30	17.6
Student	11	6.5
*Marital status*		
Married	100	58.8
Single	61	35.9
Widower	5	2.9
Separated/divorced	4	2.4
*IBD* ^3^ *type*		
UC ^4^	69	40.6
CD ^5^	101	59.4
*Smoker*		
Yes	37	21.8
No	133	78.2
*Activity index*		
Remission	92	54.1
Mildly active	60	35.3
Moderately active	18	10.6
*IBDDI category according to the score of Leong et al.* [16]		
Minimum intensity	115	67.6
Mild intensity	35	20.6
Moderate intensity	12	7.1
Severe intensity	8	4.7
*Progression of disease according to Bernklev et al.* [24]		
Less than 5 years	39	22.94
5 years or more	131	77.06
*Progression of disease according to Jägulth et al.* [25]		
Less than or equal to 2 years	25	14.71
3–4 years	14	8.24
5 years or more	131	77.06

^1^ Inflammatory Bowel Disease Disability Index; ^2^ Inflammatory Bowel Disease Questionnaire-32; ^3^ inflammatory bowel disease; ^4^ ulcerative colitis; ^5^ Crohn’s disease.

**Table 2:** Factorial structure of the Inflammatory Bowel Disease Disability Index after Varimax rotation.

Factor	1	2	3	4	5	6
**Explained variance (%)**	26.121	8.444	8.205	6.924	5.900	4.648
**Items**						
1	0.697					
2	0.777					
3	0.823					
4	0.801					
5	0.794					
6	0.723					
7	0.707					
8	0.631					
9	0.608					
10	0.748					
11	0.785					
12	0.803					
13	0.429					
14		0.735				
15		0.628				
16		0.694				
17		0.778				
14			0.651			
15			0.727			
16			0.768			
17			0.787			
b515 (lost weight)				0.585		
s540				0.668		
18					0.820	
19					0.831	
b525						0.494
b515 (BMI ^1^)						0.502
s770						0.646

^1^ Body mass index.

**Table 3:** Inflammatory Bowel Disease Disability Index contrast with sociodemographic and clinical (categorical) variables.

Variables	Mean ± SD	Median	CI (95%)	*p*-Value
*Sex*				
Male	−1.25 ± 14.01	0.00	−4.22; 1.72	0.018
Woman	−6.77 ± 15.62	−5.50	−10.20; −3.34	
*Level of schooling*				
Basic education, primary or none	−8.54 ± 17.83	−6.50	−16.07; −1.01	0.398
Middle or secondary education	−3.53 ± 14.50	0.00	−8.95; 1.88	
Complete secondary or secondary education	−2.94 ± 15.90	0.00	−6.97; 1.10	
University education	−3.19 ± 12.79	−1.50	−6.68; 0.31	
*Employment status*				
Working	−3.08 ± 15.49	−1.50	−6.28; 0.13	0.538
Unemployed	−5.08 ± 14.37	−1.00	−9.87; −0.29	
Retired	−6.27 ± 13.67	−4.50	−11.37; −1.16	
Student	−0.55 ± 17.39	3.00	−12.23; 11.14	
*Marital status*				
Married	−5.29 ± 16.25	−2.00	−8.52; −2.06	0.597
Single	−1.66 ± 13.17	0.00	−5.03; 1.72	
Widower	−2.00 ± 11.72	−4.00	−16.56; 12.56	
Separated/Divorced	−6.25 ± 12.60	−6.50	−26.31; 13.81	
*IBD* ^1^ *type*				
UC ^2^	−0.94 ± 12.82	2.00	−4.02; 2.14	0.028
CD ^3^	−5.94 ± 16.10	−3.00	−9.12; −2.76	
*Smoker*				
Yes	−5.68 ± 14.18	−5.00	−10.40; −0.95	0.232
No	−3.42 ± 15.26	0.00	−6.04; −0.80	
*Activity index*				
Remission or inactivity phase	−1.40 ± 13.61	0.00	−4.22; 1.42	0.001 ^4^
Mildly active disease phase	−2.65 ± 13.84	−0.50	−6.23; 0.93	
Moderately active disease phase	−20.94 ± 15.46	−18.00	−28.63; 13.25	
*Progression of disease according to Bernklev et al.* [24]				
Less than 5 years	−2.03 ± 14.58	−1.00	−6.75; 2.70	0.480
5 years or more	−4.47 ± 15.15	−1.00	−7.09; −1.85	
*Progression of disease according to Jägulth et al.* [25]				
Less than or equal to 2 years	−0.56 ± 13.97	−1.00	−6.33; 5.21	0.596
3–4 years	−4.64 ± 15.80	−5.50	−13.77; 4.48	
5 years or more	−4.47 ± 15.15	−1.00	−7.09; −1.85	

^1^ Inflammatory bowel disease; ^2^ ulcerative colitis; ^3^ Crohn’s disease. ^4^ The differences between groups were the following: “Remission phase” versus “Mildly active disease” (*p* = 0.695); “Remission phase” versus “Moderately active disease” (*p* < 0.001); “Mildly active disease” versus “Moderately active disease” (*p* < 0.001).

**Table 4:** Inflammatory Bowel Disease Disability Index linear regression model for men.

Variables	Coefficient	Standard Error	*t*	*p*-Value	VIF ^1^
*Constant*	−57.299	5.989	9.567	<0.001	
*Age at diagnosis*	−0.041	0.068	0.601	0.549	1.542
*Smoker*					1.066
Yes	Reference				
No	6.096	1.926	3.165	0.002	
*IBDQ-32* ^2^	0.296	0.027	10.937	<0.001	1.367
*Activity index*					1.639
Remission	Reference				
Mildly active disease	2.372	1.897	1.251	0.214	
Moderately active disease	−8.212	3.294	2.493	0.014 ^3^	
*Employment status*					1.674
Working	Reference				
Unemployed	−3.136	2.182	1.437	0.154	
Retired	−3.4832	2.156	1.615	0.110	
Student	0.389	3.066	0.127	0.899	
*Type of disease*					1.293
Ulcerative colitis	Reference				
Crohn’s disease	1.722	1.789	0.963	0.338	

^1^ Variable inflation factor; ^2^ Inflammatory Bowel Disease Questionnaire-32; ^3^ global *p*-value for activity index = 0.011 (calculated by comparing models with and without this variable). Note (summary of the model and conditions of fit): *F* = 26.03; standard error = 7.167; *p* < 0.001; *R*^2^ = 0.75; *R*^2^ adjusted = 0.72; linearity of independent quantitative variables verified by graphing the added variable; absence of collinearity verified with VIF. Normality of errors: Shapiro–Wilk test with value of *p* = 0.907; homoscedasticity: Breusch–Pagan test with *p* = 0.941. The model was adjusted to the normality criterion of the errors and homoscedasticity after eliminating three outliers that were detected after making the QQ-plot.

**Table 5:** Inflammatory Bowel Disease Disability Index linear regression model for ulcerative colitis.

Variables	Coefficient	Standard Error	*t*	*p*-Value	VIF ^1^
*Constant*	−53.764	6.621	8.119	<0.001	
*Age at diagnosis*	−0.015	0.061	0.249	0.804	1.433
*Smoker*					1.091
Yes	Reference				
No	2.155	2.760	0.781	0.438	
*IBDQ-32* ^2^	0.289	0.027	10.669	<0.001	1.543
*Activity index*					1.516
Remission	Reference				
Mildly active disease	1.691	1.469	1.151	0.254	
Moderately active disease	−4.770	4.172	1.143	0.257	
*Sex*					1.203
Man	Reference				
Woman	−1.432	1.534	0.933	0.354	
*Employment status*					1.427
Working	Reference				
Unemployed	3.899	1.834	2.126	0.037 ^3^	
Retired	−4.586	2.019	2.271	0.027	
Student	−1.663	4.349	0.383	0.703	

^1^ Variable inflation factor; ^2^ Inflammatory Bowel Disease Questionnaire-32; ^3^ global *p*-value for employment status = 0.011 (calculated by comparing models with and without this variable). Note (summary of the model and conditions of fitting): *F* = 26.49; standard error = 5.680; *p* < 0.001; *R*^2^ = 0.79; *R*^2^ adjusted = 0.76; linearity of independent quantitative variables verified by graphing the added variable; absence of collinearity verified with variation inflation factor (*). Normality of errors: Shapiro–Wilk test with *p* = 0.981; homoscedasticity: Breusch–Pagan test with *p* = 0.544. The model was adjusted to the normality criterion of the errors and homoscedasticity after eliminating three outliers that were detected after making the QQ-plot.

## Supplementary Materials

The following are available online at https://www.mdpi.com/1660-4601/16/4/635/s1, Supplementary Materials: Inflammatory Bowel Disease Disability Index (Spanish Version).
