# Supplementary material for: Cultural Adaptation and Validation of the Inflammatory Bowel Disease Disability Index in a Spanish Population and Its Association with Sociodemographic and Clinical Factors
Source: Int J Environ Res Public Health. 2019 Feb 21;16(4):635. doi: 10.3390/ijerph16040635 (PMC6406346; doi:10.3390/ijerph16040635)
Supplement: Supplementary file 1 [file ijerph-16-00635-s001.pdf]

Table S1. Inflammatory Bowel Disease Disability Index (Spanish Version).

**POR FAVOR LEA EN VOZ ALTA ESTAS INSTRUCCIONES AL PACIENTE. La primera pregunta es sobre el estado de salud del paciente, incluyendo tanto la salud física como la salud mental.**	
**RESPUESTAS: 1= Muy buena; 2= Buena; 3= Regular; 4=Mala; 5= Muy mala**	
**Estado de salud**	
1. ¿En general, cómo calificaría su estado de salud hoy?	
**POR FAVOR LEA EN VOZ ALTA ESTAS INSTRUCCIONES AL PACIENTE**<br>Ahora me gustaría examinar las diferentes funciones de su cuerpo y actividades de su vida diaria. Al responder a estas preguntas, piense en la última semana, teniendo en cuenta tanto los días buenos como los malos. Cuando le pregunte sobre una dificultad o un problema, me gustaría que valorase el grado de dificultad o problema que ha tenido mientras realizaba esa actividad de la forma en que normalmente usted la realiza. Por dificultad, me refiero a que usted necesite un mayor esfuerzo, que tenga malestar o dolor, que el desarrollo de la actividad sea más lento o haya otros cambios. Por favor, conteste a esta pregunta teniendo en cuenta cualquier ayuda de la que disponga.<br>(Lea y muestre la escala al encuestado)	
**RESPUESTAS: 1= Ninguna; 2= Leve; 3= Moderada; 4= Intensa; 5= Extrema**	
**Sueño y energía**	
2. En general, durante la última semana ¿cuál ha sido la intensidad de los problemas que ha tenido para dormir, como conciliar el sueño, despertarse frecuentemente durante la noche o despertarse muy temprano por la mañana?	
3. En la última semana ¿con qué intensidad no se ha sentido descansado y despejado durante el día? (p. ej. sentirse fatigado, sin energía…)	
**Sentimientos**	
4. En la última semana ¿con qué intensidad se ha sentido triste, bajo de ánimo o deprimido?	
5. En general durante la última semana ¿con qué intensidad ha sentido preocupación o ansiedad?	
**Imagen corporal**	
6. En general durante la última semana ¿cuál ha sido la intensidad de los problemas que ha tenido con respecto al aspecto de su cuerpo o partes de él?	
**Dolor**	
7. En general durante la última semana ¿cuál ha sido la intensidad de las molestias o dolores de estómago o abdomen que ha tenido?	
**RESPUESTAS: 1= Ninguna; 2= Leve; 3= Moderado; 4= Intensa; 5= Extremo o imposible de hacer**	
**Control de la defecación**	
8. En general durante la última semana ¿qué grado de dificultad ha tenido para regular y controlar la defecación incluyendo la elección, acceso a un lugar apropiado y llegar hasta él, así como la acción de limpiarse?	
**Cuidado de la propia salud**	
9. En general durante la última semana ¿qué grado de dificultad ha tenido para cuidar de su salud, incluyendo el mantenimiento de una dieta equilibrada?	
**Actividades de relación interpersonal**	
10. En general durante la última semana ¿qué grado de dificultad ha tenido con las relaciones personales?	
11. En general durante la última semana ¿qué grado de dificultad ha tenido para participar en la comunidad o su entorno?	
**Trabajo y estudios**	
12. En general durante la última semana ¿qué grado de dificultad ha tenido con su trabajo o las tareas del hogar?	
13. En general durante la última semana ¿qué grado de dificultad ha tenido con el colegio o sus actividades de estudio?	

▪ (b525) Número de heces líquidas o muy blandas en la última semana =<br>▪ (b515) Índice de masa corporal =<br>▪ (b515) ¿Considera que ha perdido peso en la última semana? SI/NO<br>▪ (s540) Sangre en heces (media semanal) NADA/POCO/MUCHO<br>▪ (s770) ¿Presenta artritis o artralgia? SI/NO	
**Por favor, puntúe el grado en que los siguientes aspectos del entorno del paciente influyeron de forma positiva o negativa en la actividad de la enfermedad, en las funciones corporales y en las actividades de la vida diaria**	
**RESPUESTAS: NA= No aplicable; 1= Sin efecto positivo; 2= Efecto positivo leve; 3= Efecto positivo moderado; 4= Efecto positivo intenso; 5= Efecto positivo extremo**	
14. En general durante la última semana ¿la medicación que tomó el paciente contribuyó a aliviar sus problemas y dificultades?	
15. En general durante la última semana ¿los alimentos que tomó el paciente contribuyeron a aliviar sus problemas y dificultades?	
16. En general durante la última semana ¿la familia del paciente contribuyó a aliviar sus problemas y dificultades?	
17. En general durante la última semana ¿los profesionales de la salud contribuyeron a aliviar sus problemas y dificultades?	
**RESPUESTAS: NA= No aplicable; 1= Sin efecto negativo; 2= Efecto negativo leve; 3= Efecto negativo moderado; 4= Efecto negativo intenso; 5= Efecto negativo extremo**	
14. En general durante la última semana ¿la medicación que tomó el paciente contribuyó a empeorar sus problemas y dificultades?	
15. En general durante la última semana ¿los alimentos que tomó el paciente contribuyeron a empeorar sus problemas y dificultades?	
16. En general durante la última semana ¿la familia del paciente contribuyó a empeorar sus problemas y dificultades?	
17. En general durante la última semana ¿los profesionales de la salud contribuyeron a empeorar sus problemas y dificultades?	
**RESPUESTAS: 1= No; 2= Sí**	
**Seguridad Social y servicios, sistemas y políticas de salud**	
18. ¿La Seguridad Social brinda al/ a la paciente el apoyo que necesita?	
19. ¿El paciente recibe la atención sanitaria que necesita?	
